# The effects of ipsilateral tilt position on right subclavian venous catheterization: study protocol for a prospective randomized trial

**DOI:** 10.1186/s13063-018-2666-8

**Published:** 2018-05-24

**Authors:** Dhong Eun Jung, Hyung-Chul Lee, Hyun-Kyu Yoon, Hee-Pyoung Park

**Affiliations:** Department of Anesthesiology and Pain Medicine, Seoul National University Hospital, 101 Daehak-ro, Jongno-gu, Seoul, 03080 Republic of Korea

**Keywords:** Subclavian vein, Cross-section area, Central venous catheterization, Lateral tilt

## Abstract

**Background:**

The cross-sectional area of the subclavian vein (csSCV) is an important factor determining the success rate of SCV catheterization. The head-down position increases the csSCV. However, the effects of lateral tilting on subclavian venous cross-sectional area have not yet been explored. In this trial, we test our hypothesis that ipsilateral tilt during right SCV catheterization may significantly increase the csSCV by impeding blood flow to the heart, thereby increasing the primary venipuncture success rate and reducing the complication rate and procedure time.

**Methods/design:**

This is a two-staged, prospective, randomized, controlled trial conducted on 237 neurosurgical patients requiring SCV catheterization. Seventeen patients in stage I will be placed in supine, 20° ipsilateral tilt, and 20° contralateral tilt positions in random order. The right csSCV will be measured using ultrasonography at each position. In stage II, 220 patients will be randomly assigned to the ipsilateral tilt group (*n* = 110) and supine group (*n* = 110) according to the position for right SCV catheterization. Data on catheterization-related characteristics and complications will be collected during and after catheterization. The primary outcome measures are the right csSCV for stage I and primary venipuncture success rate for stage II. The secondary outcome measures for stage II are time to venipuncture, total catheterization time, first-pass success rate, and complications, such as arterial puncture, hematoma, pneumothorax, air embolism, and catheter misplacement.

**Discussion:**

This is the first trial to investigate the effects of the ipsilateral tilt position on right SCV catheterization. We will attest the beneficial effects of the ipsilateral tilt position on the csSCV and the primary venipuncture success rate during right SCV catheterization. Furthermore, comparisons of the first-pass success rate, complications, and total catheterization time during SCV catheterization in the ipsilateral tilt position vs. the supine position will help us determine which position is better for safe and easy SCV catheterization.

**Trial registration:**

ClinicalTrials.gov, ID: NCT03296735. Registered on 25 September 2017 for stage I; NCT03303274 Registered on 6 October 2017 for stage II.

**Electronic supplementary material:**

The online version of this article (10.1186/s13063-018-2666-8) contains supplementary material, which is available to authorized users.

## Background

Central venous catheterization has been utilized in operating rooms and intensive care units for various purposes, including central venous pressure monitoring, fluid and drug administration, and parenteral nutrition. The internal jugular, subclavian, and femoral veins are used for vascular access. Central venous catheterization through the subclavian vein (SCV) is more comfortable for the patient, with a lower risk for infection but higher risk for pneumothorax compared to femoral and internal jugular venous catheterization [1–3].

The cross-sectional area of the SCV (csSCV) is an important determinant of the success rate of SCV catheterization. Body position can affect the csSCV. Previous studies have shown that the Trendelenburg position increases csSCV [4–7], while contralateral head rotation and placement of a shoulder-roll decreases it [8, 9]. However, the effects of lateral tilt on the csSCV have not been explored. Ipsilateral tilting may expand subclavian venous cross-sectional area by impeding blood flow to the heart, whereas contralateral tilting may reduce the area by venous collapse. Therefore, we hypothesized that the ipsilateral tilt position during right SCV catheterization might significantly increase the csSCV and thereby increase the primary venipuncture success rate and reduce the complication rate and procedure time compared to the supine position.

In this trial, we will compare the right csSCV among supine, ipsilateral tilt, and contralateral tilt positions to determine the beneficial effects of the ipsilateral tilt position on the csSCV, and will assess the venipuncture success rate at the first needling attempt, number of needling attempts for venipuncture, venipuncture time, total catheterization time, first-pass success rate, and complication rate during right-sided SCV catheterization in the supine position vs. the ipsilateral tilt position to determine which position is a better position for safe and easy SCV catheterization.

## Methods

### Study design

**Fig. 1:**
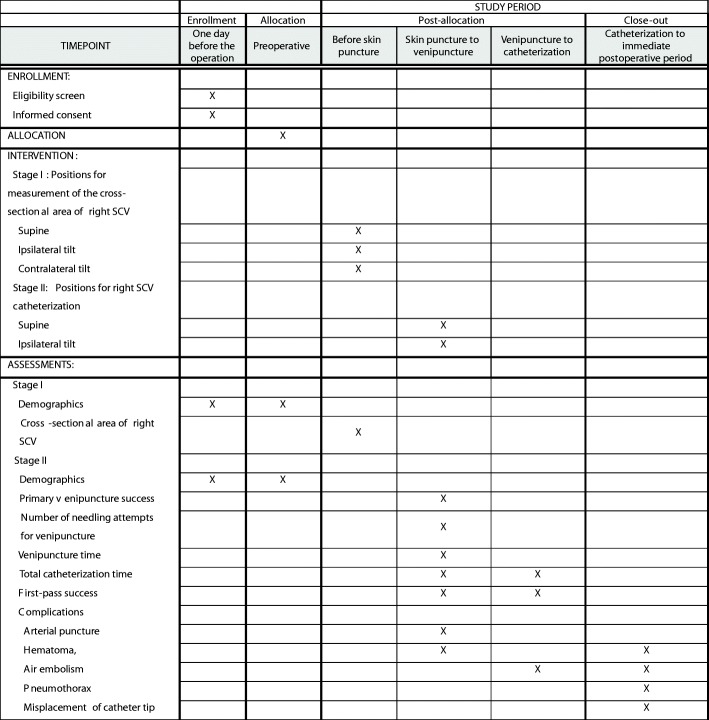
Standard Protocol Items: Recommendations for Interventional Trials (SPIRIT) flow diagram: schedule of enrollment, interventions and assessments. *SCV* subclavian vein

This is a two-staged, prospective, single-blind, single-center, superiority, randomized controlled trial. Stage I has a crossover design and stage II has a parallel randomized assignment. This study was approved by the Institutional Review Board of Seoul National University Hospital (number: 1707–110–871, date: 11 September 2017) and registered at ClinicalTrials.gov (NCT03296735 for Stage I and NCT03303274 for stage II). This trial is still being conducted at Seoul National University Hospital. The study protocol is reported according to the Standard Protocol Items: Recommendations for Interventional Trials guidelines (Fig. 1 and Additional file 1). In addition, the final report of this trial will adhere to the Consolidated Standards of Reporting Trials (CONSORT) Statement.

### Study population

Adult patients (aged 20–79 years), who are scheduled to undergo elective craniotomy to remove a brain tumor and who require SCV catheterization during the surgery, will be enrolled in this trial. The exclusion criteria are patient refusal; skin infection at the needle puncture site; a known SCV abnormality, such as an anatomical anomaly or thrombus in the SCV; tricuspid valve vegetation; anticoagulant administration; chemoport or pacemaker in situ in the SCV; having undergone right mastectomy or pneumonectomy.

### Ethics, consent, and permission

This study follows the tenets of the Declaration of Helsinki, and written informed consent will be obtained from all participants 1 day before the surgery.

### Withdrawal, dropout, and discontinuation

Participants will be withdrawn from the trial and excluded from the data analysis if they voluntarily withdraw informed consent at any time during the study period. The trial will be discontinued if serious adverse events, such as intractable cardiac arrhythmia, arterial puncture, and evident pneumothorax, and massive air embolism, occur in any patient during the procedure. Any unpredicted adverse events that occurred during the trial will be reported to the Institutional Review Board. The data analyses will be conducted according to the intention-to-treat principle.

### Randomization

**Fig. 2:**
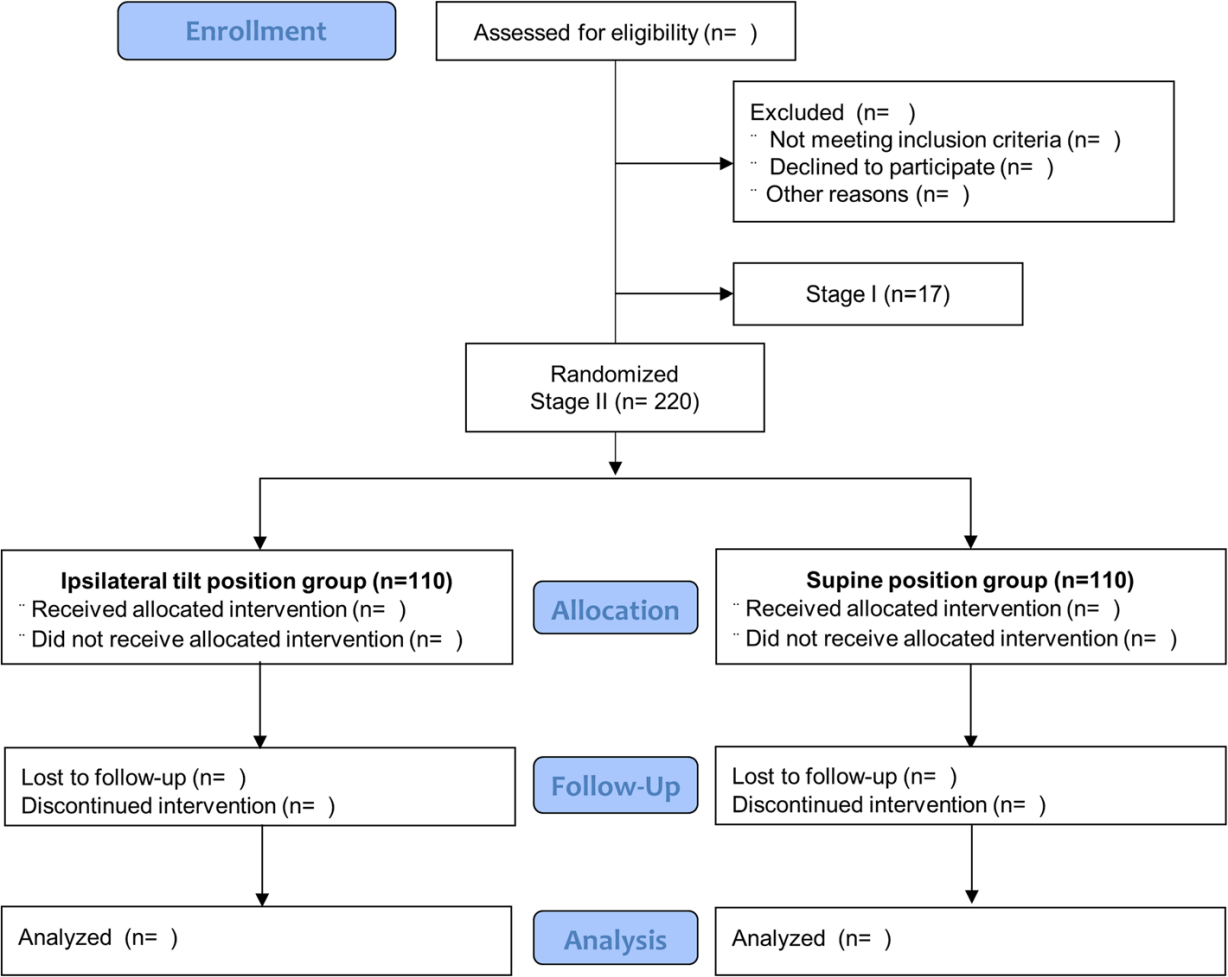
Consolidated Standards of Reporting Trials (CONSORT) flow chart

The trial follows the CONSORT flow chart from randomization to analysis (Fig. 2). The randomization tables used for stage I and stage II will be prepared before patient enrollment by an anesthesiologist blinded to this trial. An anesthetic nurse who is not involved with the trial will maintain the randomization tables in sealed opaque envelopes. Based on the order of the randomization table, the anesthetic nurse will notify the position sequence in stage I and group allocation in stage II to investigators after eligible patients enter the operating room.

In stage I with a crossover design, the patients will be placed in three different positions (supine, ipsilateral tilt, and contralateral tilt) and the right csSCV will be measured at each position. A random sequence for the three positions is made using the “RAND function” in Excel.

In stage II, patients will be randomly assigned to two groups (supine and ipsilateral tilt groups) with a 1:1 ratio according to the type of position for right SCV catheterization. A random sequence with 4 or 6 random block sizes is created using online random allocation software (http://mahmoodsaghaei.tripod.com/Softwares/randalloc.html).

### Blinding and confidentiality

This is a single-blind trial. Therefore, attending anesthesiologists (HCL and HPP) collecting the data and practitioners performing SCV catheterization are aware of the randomized position order in stage I and the group allocation in stage II.

All patients will be assigned as a study identification number, and the study data will be collected under this number. None of the information that reveals the patient’s identity, including name, social security number, and hospital chart number will be recorded. The data will be kept confidential until the data analyses are completed by the analyzers (DEJ and HKY). After study completion, the data will be stored for 3 years and then discarded according to the Korean Enforcement Rule of Bioethics and Safety Act.

### Study protocol

The first 17 patients enrolled in the study will be subjected to stage I, in which the right csSCV will be measured in the supine, 20° ipsilateral tilt, and contralateral tilt positions. After inducing general anesthesia, the patients will be placed in the three different positions based on the order predetermined by the randomized table. The tilted positions will be achieved by tilting the operating tables by 20° ipsilaterally or contralaterally. The patient’s head and body will be placed in a neutral position in the supine as well as in the two tilted positions. Each position will be maintained for 1 min and the right csSCV will be measured using a 4.5–8-MHz linear ultrasound probe (Vscan Extend, GE Healthcare, Seoul, South Korea) at the mid-point of the right clavicle. The SCV is identified by loss of pulsatility, compressibility, and a change in the area corresponding to respiration. An image of the right csSCV will be captured at the end of expiration, and the area will be measured using image-processing software (ImageJ, National Institutes of Health, Bethesda, MD, USA).

The next 220 patients will be subjected to stage II and randomly placed in either the supine or 20° ipsilateral tilted position for at least 1 min for right SCV catheterization. The head and body will remain neutral in the supine and ipsilateral tilted positions without slight Trendelenburg down. Surgical belts will be tightly strapped around the flanks and knees in the 20° ipsilateral tilted position. In addition, assistants will hold the head and body to prevent an ipsilateral head tilt and the risk of falling down, respectively. No manipulation, including placement of a shoulder-roll or pulling of the ipsilateral arm, will be used for SCV catheterization. After anesthetic induction, the right SCV catheterization will be performed using the Seldinger technique and the anatomical landmark-based method by anesthesiologists who have experienced at least 50 cases of SCV catheterization. The skin area around the right clavicle is disinfected with a solution (72% alcohol plus 2% chlorhexidine). A large, adhesive, disposable drape is aseptically applied to prevent migration of the drape from the procedural field and maintain sterility throughout the procedure. Thereafter, patients are placed in the supine or 20° ipsilateral tilted position for SCV catheterization. The skin puncture point is 1 cm lateral and 1 cm inferior from the inferior border at the mid-point of the clavicle. The skin is punctured with an introducer needle and the needle is advanced towards the SCV for venipuncture. When venipuncture is confirmed with regurgitation of venous blood into the syringe attached to the needle, a guidewire is advanced through the introducer needle. A dilator is advanced through the guidewire and removed to provide easy advancement of a 7 Fr double-lumen central venous catheter (Arrow International Inc., Reading, PA, USA). SCV catheterization is completed when the central venous catheter is inserted at a depth of 13–16 cm, the guidewire is removed, and the indwelling catheter is secured. Patients will receive positive pressure mechanical ventilation before skin puncture with the needle. Mechanical ventilation will be stopped at the time of skin puncture and then resumed after insertion of the indwelling catheter.

If the first attempt at venipuncture fails, the needle is slowly retreated until the needle tip is in subcutaneous tissue. The angle and depth of the needle will be adjusted for the next needle passage, which is dependent on the practitioner’s judgment. If the first practitioner fails to puncture the SCV after three needling attempts, the second practitioner will attempt venipuncture. If the second practitioner also fails at venipuncture after three needling attempts, the case will be classified as a failed SCV catheterization. In addition, if arterial puncture or air aspiration is evidently detected during the procedure, failure of the SCV catheterization will be considered. In cases of failed SCV catheterization, the central venous catheter will be placed in another central vein, such as the internal jugular or femoral vein.

SCV catheterization-related data, such as the number of needling attempts for venipuncture and total catheterization time, will be collected during the procedure. Clinical signs of air embolism, such as abruptly decreased end tidal carbon dioxide concentration and blood pressure, will be noted. Hematoma, a complication of catheterization, will be detected by the attending anesthesiologists primarily through inspection and manipulation of the puncture site and using ultrasonography if necessary. A chest radiograph will be obtained immediately after surgery to detect catheterization-related complications, such as pneumothorax and misplacement of the catheter tip.

### Measurements

Demographic data will be collected before SCV catheterization in the stage I patients. The right csSCV will be measured in the supine, 20° ipsilateral tilt, and contralateral tilt positions.

SCV catheterization-related data and demographics will be collected from the stage II patients. The data collected includes the success rate of SCV puncture at the first needling attempt (primary venipuncture success rate), the number of needling attempts for successful venipuncture, venipuncture time (time from skin puncture to venous blood aspiration), total catheterization time (time from skin puncture to completion of indwelling catheter insertion), first-pass success rate of catheterization (success rate of catheterization at the first attempt without any complications and technical disturbances, such as failed venous puncture or difficulties inserting the guidewire, dilator, or catheter), overall success rate of catheterization, the rate of puncture on the anterior wall of the SCV, the incidence of rescue mechanical ventilation, and the number of manipulation attempts to insert the guidewire, dilator, and catheter.

Data on catheterization-related complications, including arterial puncture, hematoma, pneumothorax/hemothorax, air embolism, and misplacement of the catheter tip (placement of the catheter tip in a location other than the superior vena cava or right atrium), will be collected during and after the procedure.

### Study endpoints

The primary endpoint measures are the csSCV in stage I and the primary venipuncture success rate in stage II. The secondary endpoint measures in stage II are the number of needling attempts for successful venipuncture, venipuncture time, total catheterization time, first-pass success rate, and complication rate.

### Sample size

The right csSCV in the supine position for stage I was reported to be 0.93 ± 0.17 cm^2^ in a previous study [10]. At an alpha level of 0.017 and a beta level of 0.2, a minimum of 15 patients are required to confirm a 15% increase in the csSCV in the ipsilateral tilt position compared to the supine position in the same patient. Considering a 10% dropout rate, 17 patients are needed for stage I.

In stage II, the primary venipuncture success rate has been reported to be 74.5% in the supine position during right SCV catheterization [11]. At the alpha level of 0.05 and a beta level of 0.2, at least 100 patients per group are needed to determine a 15% increase in primary venipuncture success rate in the ipsilateral tilt position. Assuming a 10% dropout rate, 110 patients are needed in each group.

### Statistical analysis

Statistical analyses will be performed with SPSS 21.0 (SPSS Inc., Chicago, IL, USA) and Med Calculator (MedCalc Software, Ostend, Belgium). All continuous variables will be expressed as mean ± standard deviation or median (interquartile range) based on results of the Shapiro-Wilk test. All categorical variables will be expressed as numbers (percent).

In stage I, the cross-sectional areas in the three different positions will be compared to the paired *t* test or Wilcoxon signed-rank test according to the results of the Shapiro-Wilk test. The alpha level of 0.017 (0.05/3) is set to compensate for multiple comparisons (× 3). In stage II, the number of needling attempts for venipuncture, venipuncture time, and total catheterization time between the two groups will be analyzed with the Student’s *t* test or the Mann-Whitney test. The primary venipuncture success rate, first-pass success rate, and complication rate will be analyzed with the chi-square test or Fisher’s exact test. A *P* value < 0.05 is considered significant.

## Discussion

This is the first trial to investigate the effects of ipsilateral tilt position on right SCV catheterization by comparing the csSCV and catheterization-related characteristics between the ipsilateral tilt position and the supine position.

Body position can change the csSCV. The Trendelenburg position increases the csSCV [4–7], whereas contralateral rotation of the head and placement of a roll padding under the shoulder decreases it [8, 9]. However, the effects of ipsilateral tilt position on the csSCV during right SCV catheterization have not been clarified. In this trial, we hypothesized that the ipsilateral tilt position during right SCV catheterization would expand the csSCV by impeding blood flow to the heart. We will test our hypothesis by comparing the right csSCV among three different positions (supine, ipsilateral tilt, and contralateral tilt positions).

The csSCV is an essential factor for successful SCV catheterization, particularly when the anatomic landmark-based technique is used. A large csSCV leads to a high success rate of SCV puncture at the first needling attempt, which can increase the first-pass success rate of SCV catheterization and decrease the chance of catheterization-related complications, such as arterial puncture, hematoma and pneumothorax, due to repeated needling. We will compare the primary venipuncture success rate, first-pass success rate, catheterization-related complications, and total catheterization time during right SCV catheterization between the supine and ipsilateral tilt positions to verify the clinical significance of the right csSCV on SCV catheterization.

With respect to mechanical ventilation during central venous catheterization, there is no well-defined consensus. Pneumothorax is a common SCV catheterization-related complication. A recent study showed that the incidence of pneumothorax during infraclavicular SCV catheterization was similar between patients with positive pressure ventilation and those without [11]. In contrast, another study reported that positive pressure ventilation was associated with the high risk of pneumothorax [12]. In this study protocol, mechanical ventilation will be paused during SCV catheterization to minimize the risk of pneumothorax.

In general, the slight-Trendelenberg-down position is often recommended to engorge the subclavian vein and decrease the risk of air embolism during SCV catheterization. In this study, however, SCV catheterization will be carried out in the supine and ipsilateral tilt position without slight Trendelenberg down. Because patients undergoing craniotomy for brain tumor removal are enrolled in this study, we will need to eliminate the possibility of an increase in intracranial pressure associated with the slight-Trendelenberg-down position.

Placing a patient in the ipsilateral tilt position using the operating table is a simple maneuver that can be easily done in an operating room. In this trial, we expect that the ipsilateral tilt position may be advantageous over the supine position during right SCV catheterization in terms of the success rate of catheterization, procedure time, and catheterization-related complications.

Two problems are expected when performing right SCV catheterization in the ipsilateral tilt position: there is a risk of falling and a tendency of the head to tilt towards the ipsilateral tilt position. Surgical belts strapped around the body of the patient and assistance to place the body and head of the patient in the neutral position are additionally needed to prevent such events. For the ipsilateral tilt group, we additionally pay attention to the attachment of a sterile disposable drape to maintain sterility throughout the procedure. After 20° ipsilateral tilt, the skin puncture site tends to fall below a practitioner’s comfort zone. Therefore, the surgical table is elevated until the practitioner can easily access the puncture site. Also, there is a possibility that the ipsilateral tilt position makes venipuncture difficult in some patients, especially those with obesity. However, SCV catheterization in obese patients is difficult even in the supine position. A subgroup analysis to evaluate the effect of body position on the venipuncture rate in obese patients may be helpful in clinical practice for increasing successful SCV catheterization.

In summary, in this trial, we will attest the beneficial effects of the ipsilateral tilt position on the csSCV and primary venipuncture success rate during right SCV catheterization. Furthermore, comparisons of the first-pass success rate, complications, and total catheterization time during SCV catheterization in the ipsilateral tilt position vs. the supine position will help clarify which position is safer and easier for SCV catheterization.

## Trial status

Patient recruitment commenced in October 2017 and is still ongoing. It is anticipated that recruitment will be completed in September 2018.

## Additional file

Additional file 1: Standard Protocol Items: Recommendations for Interventional Trials (SPIRIT) Checklist. (DOC 125 kb)

## Abbreviations

csSCV: Cross-sectional area of the subclavian vein
SCV: Subclavian vein
